# The effects of Teacrine^TM^, a nature-identical purine alkaloid, on subjective measures of cognitive function, psychometric and hemodynamic indices in healthy humans: a randomized, double-blinded crossover pilot trial

**DOI:** 10.1186/1550-2783-11-S1-P49

**Published:** 2014-12-01

**Authors:** SM Habowski, JE Sandrock, AW Kedia, Tim N Ziegenfuss

**Affiliations:** 1 The Center for Applied Health Sciences, Stow, Ohio, USA

## Background

Aside from caffeine, there is a relative dearth of evidence regarding natural ingredients that enhance subjective “energy” levels. We have studied a unique, naturally occurring purine alkaloid, present in *Camellia assamica* variety kucha tea (amongst other botanical sources) that acts on both adenosinergic and dopaminergic pathways that appears to influence multiple neurochemical pathways. As a first step in a series of experiments, we examined the effects of TeaCrine^™^, a nature-identical, chemically equivalent bioactive compound known as theacrine (1,3,7,9-tetramethyluric acid), in humans.

## Methods

Using a randomized, double-blinded, within-subject (crossover) design, 15 healthy subjects (mean ± SD age, height, wgt, BMI: 28.3 ± 6.1 y, 175.7 ± 11.5 cm, 89.8 ± 21.7 kg, 29.1 ± 4.7) volunteered to ingest 200 mg of TeaCrine^™^ (Compound Solutions, Inc., Carlsbad, CA) (TC) or Placebo (PLA). Anchored VAS questionnaires were used to detect changes in various aspects of physical and mental energy and performance; side effect profiles, hemodynamics and biochemical markers of safety were also collected over a 3-hr post-dosing period. A subset of 6 subjects underwent a separate 7-day, open-label, repeated dose study comparing 100 mg, 200 mg and 400 mg of TC. Consent to publish the results was obtained from all participants.

## Results

The 200 mg dose of TC caused significant improvements in energy (TC: +8.6% vs. PLA: -5.7%, P=0.049) and reductions in fatigue (TC: -6.7% vs. PLA: +5.8%, P=0.04). A trend for improved concentration was also noted (TC: +2.4% vs. PLA: -1.3%, P=0.07). No changes in systemic hemodynamics or side effect profiles were noted. The N=6 cohort study demonstrated moderate to large effect sizes (0.50 to 0.71) with the 200 mg dose of TC over a 7-day period of assessment for the following subjective measures: energy, fatigue, concentration, anxiety, motivation to exercise and libido.

## Conclusion

These preliminary data support the benefits of acute TeaCrine^™^ supplementation on subjective “energy” levels and some indices of mental performance. Future studies are underway to confirm these neurotropic effects and also explore potential benefits of TeaCrine^™^ on objective measures of cognitive and physical performance, inflammation, pain perception, and functional capacity.

